# Bacterial Diversity of Diabetic Foot Ulcers: Current Status and Future Prospectives

**DOI:** 10.3390/jcm8111935

**Published:** 2019-11-10

**Authors:** Fatemah Sadeghpour Heravi, Martha Zakrzewski, Karen Vickery, David G. Armstrong, Honghua Hu

**Affiliations:** 1 Surgical Infection Research Group, Faculty of Medicine and Health Sciences, Macquarie University, Sydney 2109, Australia; f.sadeghpour1989@gmail.com (F.S.H.); karen.vickery@mq.edu.au (K.V.); 2 QIMR Berghofer Medical Research Institute, Brisbane QLD 4006, Australia; Martha.Zakrzewski@qimrberghofer.edu.au; 3 Southwestern Academic Limb Salvage Alliance (SALSA), Keck School of Medicine of University of Southern California (USC), Los Angeles, CA 90007, USA; armstrong@usa.net

**Keywords:** diabetic foot ulcers, diabetic foot infections, microbiology, culture, culturomics, 16S rRNA sequencing, microbiota, metagenomics, metatranscriptomics

## Abstract

Diabetic foot ulcers (DFUs) and diabetic foot infections (DFIs) are associated with reduced patient quality of life, lower-extremity amputation, hospitalization, and high morbidity and mortality. Diverse bacterial communities have been identified in DFUs/DFIs, playing a significant role in infection prognosis. However, due to the high heterogeneity of bacterial communities colonized in DFUs/DFIs, culture-based methods may not isolate all of the bacterial population or unexpected microorganisms. Recently, high sensitivity and specificity of DNA (metagenomics) and RNA (metatranscriptomics) technologies have addressed limitations of culture-based methods and have taken a step beyond bacterial identification. As a consequence, new advances obtained from DNA- and RNA-based techniques for bacterial identification can improve therapeutic approaches. This review evaluated the current state of play in aetiology of DFUs/DFIs on culture and molecular approaches, and discussed the impact of metagenomic and metatranscriptomic methods in bacterial identification approaches.

## 1. Introduction

The number of people with diabetes is expected to increase rapidly—from 425 million in 2017 to a predicted value of 600 million by 2030. More than one-third of people with diabetes develop diabetic foot ulcers (DFUs) during their lifetime, with half of these becoming infected and causing diabetic foot infections (DFIs). Fifteen percent of patients with DFIs require lower limb amputation to prevent progression of the infection [1,2].

Diabetic foot care is very expensive, with an estimated US $8659 annual cost per patient, thus emphasizing the importance of early diagnosis and treatment of DFUs/DFIs [3]. Treatment consists of improving patient intrinsic factors, such as improving glucose control, as well as targeting extrinsic factors, the principal being the removal of bacterial contamination/infection. However, DFUs/DFIs harbor diverse bacterial communities, which increase the difficulty in treatment choice.

There are several laboratory techniques available with different sensitivities and specificities to determine the bacterial composition of DFUs/DFIs. Nonetheless, the characterization of the entire polymicrobial community at different severity stages ranging from mild to severe is still a major challenge [4].

Although culture-based methods are the principal method of bacterial identification, they often produce false-negative results in patients who have received antibiotics; fail to identify slow-growing, fastidious, anaerobic, and unknown pathogens; and are time-consuming, hindering proper and early detection of the bacterial community in DFUs/DFIs. Recent advances in molecular technologies overcome many of the mentioned inadequacies and provide new insights into the bacterial diversity of DFUs/DFIs. These advancements have important implications for the identification of so far unknown and uncultivable bacteria in DFUs/DFIs.

This manuscript will review the current state of play in culture and molecular methods to assess the bacterial diversity in DFUs/DFIs, and analyze the future impact of metagenomics and metatranscriptomic approaches on bacterial identification and treatment.

## 2. Sample Collection from DFIs

The first and most critical step, not only in culture-based methods but also in advanced molecular-based approaches, is sample collection. Historically, curettage, biopsies, swabs, and wound aspirations have been the principal routine samples taken by wound care providers [5]. As the Infection Disease Society of America (IDSA) advises that samples be taken from the base of wounds, tissue biopsies have been proposed as a gold standard method [6]. Swab cultures of the wound surface are also commonly used, but due to a high number of commensal microflora inhabiting healthy skin, swab culture results may not be as reliable as tissue samples [5]. For instance, coagulase-negative *staphylococci* (CoNS), *Micrococcus*, *Bacillus* spp., and *Corynebacterium,* which are a part of normal skin flora and have been frequently isolated from DFIs swabs, are not usually considered as pathogenic bacteria, unless the samples are taken from deep tissues [7]. Even though the collection of swab samples is easier than tissue samples, some studies have shown that swab culture results are less specific and sensitive [7,8].

Although culturing of superficial swabs and deep tissue specimens from infected ulcers provided identical results in 62% of cases, the swabs only identified 91% of the organisms isolated from tissue samples [9]. Similar results were obtained by Mutluoglu in 69.2% of wounds, but superficial swabs failing to detect all organisms in 9% of cases. The positive predictive value of swabs relative to tissue was 84.4% [10].

Swab samples are less reliable in isolating Gram-negative bacteria such as *E. coli* and *Citrobacter* [11]. A higher concordance rate of 80% was found by Huang et al. in deep ulcers; however, when abscess osteomyelitis or gangrene was present, significantly different results were obtained by swabs and tissue biopsy with only around 30% concordance. Moreover, some Gram-negative bacteria, such as *Ralstonia pickettii* and *Serratia,* were only identified in deep tissue samples [11].

Deep tissue samples also showed higher sensitivity for the monitoring of bacterial species that have been previously reported as antibiotic-resistant strains [8]. Similarly, percutaneous bone biopsy identified a higher number of organisms causing diabetic foot osteomyelitis compared to swab samples [12,13]. Significantly more bacteria were isolated from tissue samples compared to 247 paired swab samples with a 42% concordance [14].

Based on the aforementioned studies that compared the efficiency of bacterial culture using tissue and swab samples, it can be stated that tissue samples provide more reliable results for bacterial identification and monitoring of bacterial population in DFIs.

## 3. Microbiology of DFUs/DFIs

**Figure 1:**
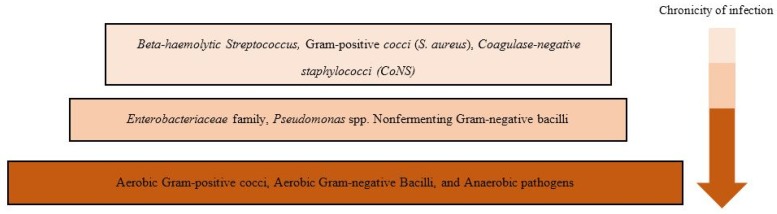
Bacterial diversity of diabetic foot infections in infection progression.

According to bacterial culture and molecular approaches, DFUs/DFIs can be colonized by different aerobes and anaerobes. DFIs of a shorter duration seem to have a simpler microbiota and are mainly colonized by Gram-positive *cocci* (*Staphylococcus* and *Streptococcus* spp.). In contrast, chronic DFIs may have polymicrobial infections colonizing by different types of aerobic bacteria, such as *Staphylococcus*, *Streptococcus*, *Enterococcus*, *Pseudomonas* spp., and anaerobic pathogens (Figure 1) [15]. *Bacteroides fragilis* has also been reported in several studies as the most abundant anaerobic bacteria in DFIs [16,17]. Based on these studies which were explicitly designed to culture anaerobes, anaerobic bacteria were reported in low abundance with low impact on infection progress.

Geographical features, infection duration, patient’s metadata (e.g., smoking habits), and antibiotic use can also influence the bacterial distribution in patients. For example, a Korean study conducted on 737 patients found that smoking can increase the risk of DFIs with *Pseudomonas* spp. [18].

### 3.1. Bacterial Identification of DFIs Based on Culture

#### 3.1.1. Gram-Positive Bacteria

**Figure 2:**
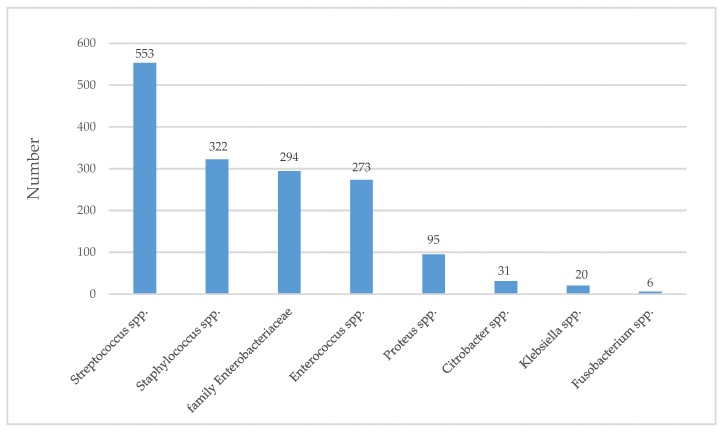
Commonly isolated bacteria from DFIs using culture-based methods. The number on the bar is the sum of the total number of isolated bacterial genus in 10 studies from 2004 to 2018.

Firmicutes is the main bacterial phylum, comprising *Streptococcus* spp. (*Streptococcus agalactiae, Streptococcus pyogenes*, and *Streptococcus mitis*), *Staphylococcus* spp. (*Staphylococcus aureus*), and *Enterococcus* spp. (*Enterococcus faecalis*) (Figure 2). *S. aureus* has been reported as the most common pathogenic species in DFIs in several studies. In a study conducted on 342 patients with diabetic foot infections, *S. aureus* (20.2% of isolates) was the most common Gram-positive bacteria [19]. These results are relatively similar to the number of Gram-positive bacteria in Jneid’s study (54.7% of isolates) [20] and Al Benwan’s study (32.3% of isolates) [21], which applied culture and culturomic methods to isolate bacterial species, respectively. *Staphylococcus epidermidis* was also isolated in one study conducted on 454 DFIs samples as the most dominant bacterial species. Although *Staphylococcus epidermidis* is part of the normal skin, it can cause severe infections in the presence of foreign bodies, such as prosthetic devices and wound infections [22,23].

#### 3.1.2. Gram-Negative Bacteria

The predominance of the *Enterobacteriaceae* family (*Escherichia coli*, *Klebsiella pneumoniae*, *Morganella morganii,* and *Proteus mirabilis*) has recently been reported as the largest group of aerobic Gram-negative rods in DFIs. For instance, an average of 1.8 bacterial pathogens per diabetic wound sample was reported in one study, of which, 51.2% were Gram-negative bacteria [21], which was quite high compared to the number of Gram-negative species in Jneid’s study (26.4% of isolates) [20]. This discrepancy might be due to previous antibiotic use in patients, long duration of hospitalization, and wound chronicity. *Escherichia coli* was also reported as the most common Gram-negative bacteria in 342 patients with diabetic foot infections [19]. *Entrobacter*, *Pseudomonas*, *Citrobacter,* and *Provetella* spp. have also been reported in lesser numbers. 

#### 3.1.3. Polymicrobial Infection

DFIs are composed of a mixture of bacteria. Consequently, the production of many different virulence factors, such as proteases, collagenases, and hemolysins contribute to infection chronicity [23]. Polymicrobial infections were estimated to occur in 75% of DFI cases in Al Benwan’s study conducted on 440 DFIs samples [21]. In Citron’s study, 83.8% of positive cultures were polymicrobial with a mixed population of Gram-positive and Gram-negative species [23]. In another study conducted on 473 specimens, 56.87% of the DFIs were polymicrobial with a high abundance of Gram-negative isolates (76.27%) [24]. The occurrence of polymicrobial infections was reported to be relatively lower in Sánchez’s study (48.3%) [25]. Polymicrobial infections are more challenging to treat compared to DFIs with simpler bacterial composition and are likely to develop complex and chronic infections [4].

**Figure 3:**
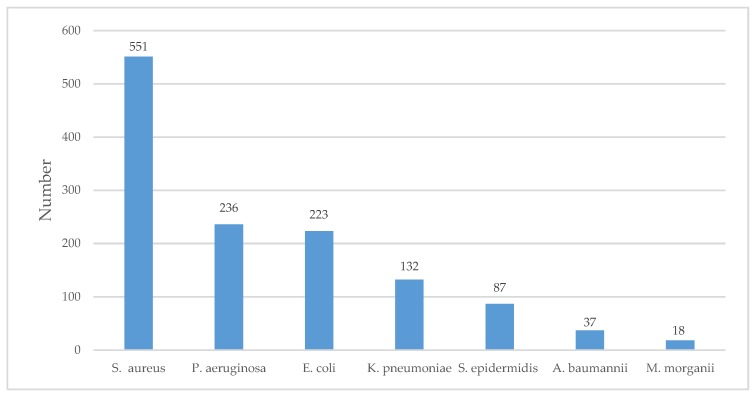
Commonly isolated bacterial species in DFIs using culture-based methods. The number on the bar is the sum of the total number of the isolated bacterial species in 10 studies from 2004 to 2018.

Figure 2 and Figure 3 show the total number of commonly isolated bacterial genus and species in DFIs from 2004 to 2018.

#### 3.1.4. Uncommon Bacterial Species

Compared to the pathogenicity of *S. aureus*, the role of uncommon isolated bacteria in DFIs is less clear. *Finegoldia magna* is an anaerobic Gram-positive *coccus*, which is part of the normal flora of the genitourinary and gastrointestinal tract and can be isolated from the oral cavity and skin.

Based on Citron’s findings, *F. magna* was isolated as the most predominant anaerobe (37.4%) [23], which has not been commonly reported by previous studies. One possible reason can be the different selective bacterial media that have been used in other studies. For instance, Brucella agar is commonly used for anaerobes cultivation, which is also a selective culture media for *B. fragilis* and *Prevotella* species, but not for Gram-positive anaerobes (such as *F. magna*). This explanation may describe why several studies have reported *B. fragilis* as the predominant anaerobes [23]. Furthermore, *Porphyromonas* and *Prevotella*, *Clostridium*, *Aerococcus, Helcococcus*, and *Gemella* spp. as fastidious and slow-growing bacteria have been rarely isolated from DFIs [26].

Although culture-based methods have been the gold standard for bacterial identification for many years, this approach may not necessarily reflect all the clinically important pathogenic bacteria in DFIs, particularly anaerobes and uncommon species.

### 3.2. Bacterial Identification of DFIs Based on Culturomic Method

Culturomics involves optimization of culture conditions using matrix-assisted laser desorption ionization-time of flight mass spectrometry (MALDI-TOF MS) to overcome culture-based method limitations. MALDI-TOF MS produces specific mass spectral fingerprints from bacterial proteins, which is a unique signature for bacteria. All the bacterial protein fingerprints are compared to a protein database in order to facilitate accurate bacterial identification to genus, and even species level [27]. Culturomics revealed that 88.3% of DFIs were polymicrobial, identifying 53 known and 19 unknown bacterial species. Based on culturomic findings, *S. aureus*, *Enterococcus faecalis*, *Enterobacter cloacae*, *Staphylococcus lugdunensis*, *Staphylococcus epidermidis*, *Proteus mirabilis,* and *F. magna* in descending order were the most abundant bacterial species isolated from the 43 patients [20].

On the downside, fresh bacterial colonies and a fair amount of bacterial biomass are needed to be detected by mass spectrometer, which may limit the use of culturomics for fastidious and slow-growing bacteria. Further evaluation of microbiota in DFIs using this method on a larger scale is required to identify the superiority of this method to other bacterial identification tools.

High specificity and sensitivity of molecular approaches have provided a more reliable insight into DFIs’ microbiota than traditional methods. The bacterial 16S ribosomal RNA (rRNA) gene presented in all bacteria is a widely used phylogenetic marker for bacterial taxonomic classification and is particularly effective for anaerobes, slow-growing, and fastidious bacteria [28].

## 4. Molecular DNA-Based Techniques

Depending on the phenotypic and genotypic characteristics of pathogenic bacteria, the initial assessment of bacterial isolation using a culture-based method may require one week or longer. Regardless of bacterial characteristics in a heterogeneous sample, molecular approaches can minimize this time frame to a few days and provide more reliable results. DNA-based approaches have cast new light on the multiplex bacterial community in DFUs/DFIs and have played a significant role in accurate bacterial identification and the evaluation of new bacterial species, and even new genes that play a significant role in infection progression. DNA-based approaches can provide not only bacterial taxonomic classification, but also antibiotic susceptibility pattern (resistome), which may help the replacement of empirical therapies with targeted therapies [7]. 

**Figure 4:**
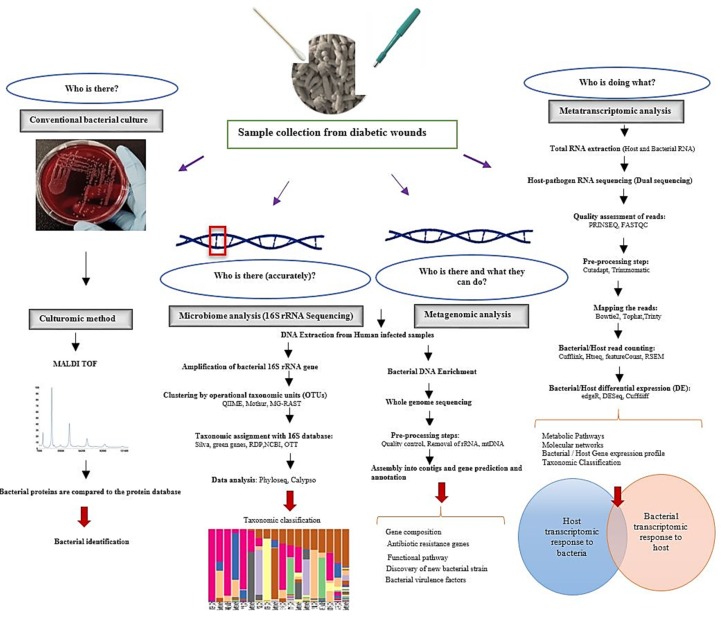
Overview of different approaches to evaluate bacterial species in infected cells.

Polymerase chain reaction (PCR), denaturing gradient gel electrophoresis (DGGE), 16S rRNA gene sequencing analysis, metagenomics (whole genome sequencing), and metatranscriptomics (RNA profiling) have provided scientists with new insights into the bacterial community structure and the alteration of the human microbiome during infection (Figure 4). Understanding the bacterial gene composition and functional capabilities of pathogenic microbes using molecular-based approaches will enable scientists to more accurately target bacterial virulence factors and enzymes associated with infection.

### 4.1. Bacterial Identification of DFUs/DFIs Based on 16S rRNA Sequencing

The 16S rRNA gene has been highly conserved through bacterial evolution. This gene consists of hypervariable regions (V1–V9), which enable the identification of different bacterial taxa [29]. A common approach to analyze 16S rRNA gene sequences is to group them into operational taxonomic units (OTUs), based on a pair-wise sequence identity of 97%, which is commonly considered as being equivalent to species level [30].

#### 4.1.1. Gram-Positive Bacteria

Comparison of traditional culture and 16S rRNA gene sequencing approaches to DFUs showed that the most abundant OTU belonged to *Staphylococcus* spp. (such as *S. aureus* and *S. epidermidis*), which may demonstrate the critical role of *Staphylococcus* spp., particularly *S. aureus,* as a pathogenic bacteria in DFUs /DFIs, which have been reported frequently in other studies that applied 16S rRNA sequencing [31,32]. In Gardner’s study [33], the culture-based method underestimated the number of bacterial species isolated from DFUs (26 bacterial species per DFU), compared to the number of species-level OTUs discovered by 16S rRNA sequencing analysis. For instance, the culture-based method showed the presence of anaerobes in 27% of the samples, while 16S rRNA sequencing identified anaerobic bacteria in all the samples (100%). The comparison of bacterial diversity on new and recurrent diabetic ulcers using 16S rRNA amplicon sequencing showed more reliable results compared to culture-based methods. The traditional bacterial culture showed positive bacterial growth in 55% of the patients, while 16S amplicon sequencing showed positive bacterial detection in 75% of the patients in new and recurrent ulcers. Bacterial culture revealed the presence of *S. aureus*, anaerobes, beta-hemolytic *streptococci*, and *Candida* spp. in descending order, while 16S rRNA sequencing analysis showed a wider range of bacterial diversity (*Peptoniphilus*, *Staphylococcus*, *Anaerococcus, Corynebacterium Corynebacterium*, *Peptoniphilus*, and *Anaerococcus*) in new and recurrent diabetic ulcers [31].

#### 4.1.2. Gram-Negative Bacteria

*Escherichia, Proteus, Klebsiella, Enterobacter, Pseudomonas, Citrobacter, Provetella, Bacteroides, Porphyromonas, Proteobacteria, Bacteroidetes*, and *Fusobacteria* spp. have been reported as the most dominant Gram-negative bacterial strains by the 16S rRNA sequencing method from DFUs/DFIs, which have also been reported using culture-based methods [31,32,33,34].

#### 4.1.3. Polymicrobial Infection

Based on 16S rRNA analysis, deep ulcers had a more diverse bacterial community being colonized by anaerobes, Gram-positive, and Gram-negative bacteria. Analysis of the 16S rRNA gene showed that superficial wounds had mainly *Staphylococcus* spp. [33]. Similarly, assessment of 2,963 different types of wounds (diabetic foot ulcers, venous leg ulcers, and decubitus ulcers) revealed a great bacterial diversity in chronic wounds with a high number of *Staphylococcus* (63%) and *Pseudomonas* (25%) species, anaerobes, and commensal bacteria [35]. Longer duration DFUs were found to be polymicrobial with an average of 10 to 125 bacterial species [36]. 

Although both culture-based methods and molecular approaches have confirmed the polymicrobial nature of DFUs/DFIs, culture-based methods may not be an appropriate method to fully evaluate this complexity. As a result, wound care management based on inaccurate bacterial identification may lead to unsuccessful antimicrobial outcomes. 

#### 4.1.4. Uncommon Species

**Figure 5:**
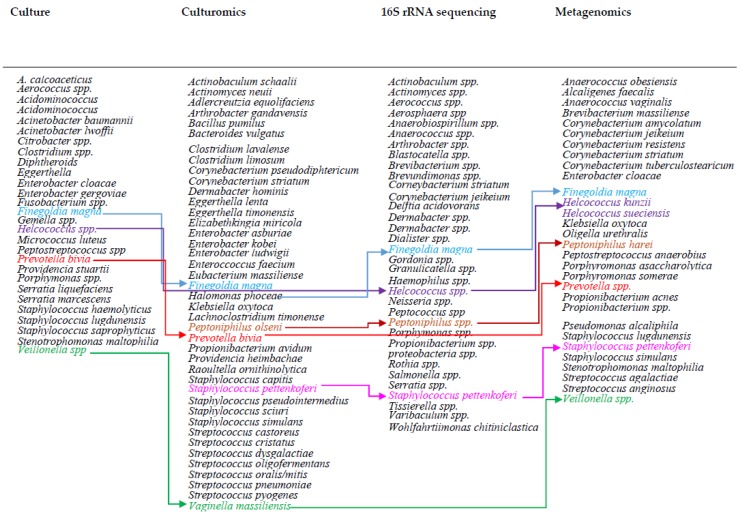
Uncommon bacterial species isolated from diabetic ulcers using different bacterial identification tools.

16S rRNA sequencing analysis has identified a wider range of uncommon bacterial species in DFUs/DFIs, compared to the culture-based method, which may indicate the inadequacy of culture-based methods in the identification of anaerobes and fastidious bacteria [37]. *Delftia acidovorans, Serratia nematodiphila, Streptococcus salivarius, Fusobacterium nucleatum, Flavobacterium succinicans, Staphylococcus pettenkoferi,* and many other species have been detected by the 16S rRNA sequencing method, which had not commonly reported by culture-based methods. Figure 5 shows uncommon bacterial species isolated from DFUs using different identification tools.

### 4.2. Bacterial Identification of DFUs Based on Shotgun Metagenomic Sequencing

In 16S ribosomal RNA (rRNA) amplicon sequencing, just a single region of the bacterial genome (16S rRNA gene) is sequenced, but shotgun metagenomic sequencing is a whole-genome sequencing approach. While the 16S rRNA gene is highly important in bacterial identification, it lacks mechanistic insights into DFUs/DFIs, such as antimicrobial susceptibility pattern, functional pathway, gene composition, biofilm, and virulence factors-related genes. Also, 16S rRNA amplicon sequencing is less sensitive at the species level. Contamination with human mitochondrial DNA (mtDNA) is another problem that can reduce microbial sequencing throughput and introduce bias in the 16S rRNA downstream analysis [30].

Shotgun metagenomic sequencing is a genome-wide sequencing approach to assess bacterial communities directly from infected sites. Shotgun metagenomic sequencing can address the limitations of culture and 16S rRNA sequencing methods and even identify unexpected microbial taxa. This relatively young, but rapidly growing field can provide a clear insight into the potential interaction of DFUs’ microbiota. It also provides useful data on phylogeny, new enzymes, biocatalysts, and the function of uncultivable and unknown microbes [38]. The rapid price reduction in next-generation sequencing and increased sequence data throughput will make shotgun metagenomic sequencing a promising approach, in the same manner as 16S amplicon sequencing [39].

#### 4.2.1. Gram-Positive Bacteria

Shotgun metagenomic sequencing of 100 non-infected diabetic foot ulcers was performed to investigate the DFUs microbial community at the strain-level resolution and to identify the virulence and pathogenicity of DFUs microbiota. *S. aureus*, *Pseudomonas aeruginosa*, and *Corynebacterium striatum* were identified as the most abundant bacterial species in this study. Also, metagenomics identified the main *Staphylococcus* strains, including *S. aureus* 7372 and *S. aureus* 10757 as dominant strains in DFUs for the first time [40].

#### 4.2.2. Gram-Negative-Bacteria

Based on shotgun metagenomic sequencing, *Alcaligenes faecalis* was the most abundant Gram-negative bacteria in DFUs, which was also previously reported by the culturomic method [20,40]. The measurement of the inflammatory response in keratinocyte cells also showed that *A. faecalis* could induce the production of cytokine IL-8 as a pro-inflammatory cytokine; and GM-CSF, G-CSF, and PDFG-AB, which can lead to wound healing enhancement [40].

#### 4.2.3. Uncommon Bacterial Species

Coagulase-negative species *Staphylococcus pettenkoferi, Staphylococcus simulans*, and *Staphylococcus lugdunensis* were reported in less abundance from DFUs using metagenomics. *Corynebacterium striatum, Propionibacterium* spp., *Porphyromonas somerae, Brevibacterium massiliense, Klebsiella oxytoca*, and many other uncommon species were also reported in DFUs using shotgun metagenomic sequencing (Figure 5) [40]. Further investigation is needed to evaluate the importance of isolated uncommon species in the chronicity and progression of DFIs.

#### 4.2.4. Functional Pathways

Based on the annotation of DFUs’ microbiota pathways using SEED [41], the pathway of carbohydrate, protein, and amino acid metabolism related genes to virulence and infection, transposable elements, and phages were the most abundant features in DFUs’ microbiota. Deep wounds with insufficient oxygen exchange were associated with the anaerobic glycolysis production system, biosynthesis of saccharide, production of capsular, and extracellular polysaccharide, which all support the presence of bacterial taxa with the property to produce a biofilm structure in deep layers of the infection site. Whole-genome sequencing of *S. aureus 7372* and *S. aureus 10757* strains showed the presence of the *agrABCD* gene, which is responsible for autoinducing peptide production and quorum-sensing system. *SA7372* was also enriched with different virulence factors related to immune evasion, such as staphylokinase (*sak*) to spread the infection, an additional copy number of *lukDV*, *lukEv* which produce neutrophil targeting leukotoxin, an additional *lytN* to coordinate synthesis of the bacterial cell wall, and *scn* genes to inhibit opsonization and phagocytosis by the human immune system [40].

Antimicrobial susceptibility testing of DFIs has mainly relied on culture and PCR-based methods and is restricted to frequently isolated bacteria and commonly used antibiotics. Sample collection and transport can also highly influence the result of antimicrobial susceptibility tests.

Identification of DFI resistome using metagenomic approaches can overcome these limitations and provide a greater amount of information regarding all the genes involved in antibiotic resistance (DFI resistome). Metagenomics can provide new chances for the investigation of novel resistance mechanisms in DFUs/DFIs and accordingly the development of existing treatment approaches. Recently metagenomics of *SA10757* strain isolated from DFUs showed the presence of different antibiotic resistance genes, such as macrolide (*ermA*), tetracycline (*tetA*), and an aminoglycoside (*ant1*), *sec2* and *sea,* which may explain the increasing prevalence of multidrug-resistant organisms in DFUs [40].

Many aspects of pathogen detection in DFUs/DFIs have improved dramatically and are summarized in recently published studies. Table 1 lists commonly reported DFUs/DFIs microbiota using different bacterial identification tools.

## 5. DFUs/DFIs Enter the Metatranscriptomic Era

While bacterial pathogens destroy human cells by cellular pathway manipulation for multiplication and survival, human cells respond to these pathogens through cascade changes in the innate and humoral immune system. Inconstancy of wound microbiota and bacterial transition patterns over time is thought to play an important role in the healing process of DFUs/DFIs [47].

RNA sequencing has emerged as an accurate technology to study metabolically active microbiota and the host-microbes transcriptome, simultaneously avoiding many drawbacks of microarrays, such as cross-hybridization, background noise, and restriction of gene identification [48].

Different types of RNA such as coding (mRNA) and non-coding RNA (microRNAs, siRNA, snoRNAs) also can be analyzed by metatranscriptomic approaches. Transcriptional responses of wounds to *P. aeruginosa* infections showed that 2 h after bacterial infection, wounds had down-regulation of ncRNAs (snoRNA, miRNA, and RNU6 pseudogenes), while 6 and 24 h after infection, wounds had down-regulation of protein-coding genes with an overrepresentation of ncRNAs prior to down-regulation of skin-enriched coding gene expression in the host. These findings suggest that regulation of different classes of ncRNAs follow a consecutive and harmonized pattern during transition state from inflammation to the healing process [48].

Furthermore, possible correlations between the expression of different virulence genes in *S. aureus* isolated from DFUs have also been identified [49]. In this regard, scientists have used oligonucleotide microarrays designed for specific encoding genes in the *S. aureus* genome to identify the bacterial genomic profile in uninfected and infected diabetic foot ulcers. A comparison of *S. aureus* expressed virulence genes in infected and uninfected ulcers showed that toxic shock syndrome toxin (*tsst*), leukocidins (*lukF*, *lukS*, *lukS-PV*, and *lukF-PV*), exfoliatins (*etA*, *etB*), and enterotoxins (*sea*, *seb*, *sec*, *sek*, *seq*) were not identified in uninfected ulcers (grade 1), but are mainly expressed in infected ulcers (grade 2–4). Additionally, a Panton-Valentine leukocidine gene (encoding β-pore-forming toxin) was present in deep ulcers (Grade 4). In a similar study, the expression profile of uninfected ulcers (grade 1) and infected ulcers (grade 2–4) was examined using conventional PCR of *cap8*, *sea*, *sei*, *lukE*, and *hlgv* genes. Isolated strains from infected ulcers were found to remarkably be more virulent than strains from uninfected ulcers. A combination of these five virulence genes may help differentiate infected ulcers from non-infected ulcers and to anticipate wound status at the follow-up [50].

This information indicates the importance of gene expression profiling during infection progression, which can improve our understanding of disease development, as well as prognosis and development of therapeutical approaches. Shotgun metagenomic sequencing and metatranscriptomics can predict the relative abundance of DFUs/DFIs microbiota, which can express a particular virulence gene based on infection severity. As this topic is still in its infancy, further investigations are warranted to evaluate if screening of wound microbial activity of specific wound bacterial species and metabolic gene expression can determine if a DFU will become infected. By using molecular analysis, more bacterial communities are predicted to be isolated from DFUs/DFIs, particularly from deep infection ulcers, which are an optimal place for the growth of anaerobes. Due to the excellent performance of molecular methods in recent years, it is likely that phenotypical methods will be replaced with DNA-based methods as frontline diagnostic tests in the future. New findings based on molecular technologies can improve traditional therapies and replace them with more evidence-based therapy.

## 6. Treatment of DFIs

While antibiotic treatment for DFIs is initially prescribed empirically, accurate bacterial identification of DFIs can improve therapeutic approaches. The selection of the most effective antibiotic is a vital step to reduce the treatment period, prevent the expansion of resistant bacterial strains, and limit health costs [51]. Methicillin-resistant *S. aureus* (MRSA) is a common bacterial pathogen in DFIs and is a difficult-to-treat infection. Compared to non-MRSA infections, diabetic people with MRSA infections have a greater than fivefold increase in mortality [52]. The susceptibility pattern of *Staphylococcus* spp. and Gram-negative bacteria should regularly be monitored in DFIs, while other organisms may be analyzed selectively [53].

In the following section, wounds have been classified based on the Infectious Disease Society of America system (IDSA).

### 6.1. Non-Infected Wounds

To prevent possible infection and improve the wound-healing process, different classes of antibiotics are being prescribed by clinicians, even for uninfected wounds. There is, however, no consensus to support this practice, which may also increase treatment-related adverse events (TRAEs) and the expansion of resistant bacteria. The European Wound Management Association has prohibited antibiotic prescription for uninfected wounds [54].

### 6.2. Mild Infections

Mild infections are characterized by inflamed subcutaneous tissue, which may vary in size but are found less than 2 cm from the surrounding of the ulcer. Gram-positive cocci, *S. aureus*, and group B *streptococci* have been frequently isolated in the mild stage. Hence, antibiotics that target Gram-positive bacteria may be a good choice for this stage of infection. Mild infections can be healed using oral antibiotics (quinolones, trimethoprim-sulfamethoxazole, and clindamycin). Based on the IDSA guideline, mild infections require a shorter course of antibiotic treatment (up to two weeks), compared to moderate and severe infections [55].

### 6.3. Moderate to Severe Infections

A heterogeneous mixture of aerobic Gram-positive cocci, aerobic Gram-negative Bacilli, and anaerobic pathogens can be isolated from moderate to severe stages of infection [56]. Based on the IDSA guideline, moderate to severe infections need up to four weeks of antibiotic therapy [55]. Narrow spectrum oral antibiotics and broad-spectrum parenteral antibiotics are administered for mild and severe infections, respectively. In either case, the presence of *Staphylococcus* and *Streptococcus* species should always be considered [51]. Cotrimoxazole, tetracyclines, rifampin, linezolid, fluoroquinolones, and clindamycin have a proper absorption rate and are effective for systemic circulation when administered orally for moderate to severe infections [57].

### 6.4. Natural Antimicrobial Peptides

Due to the high incidence of multidrug-resistant bacterial strains, traditional antibiotics have become less effective against a broad range of bacteria. Natural antimicrobial peptides (AMPs) or host defense peptides (HDPs), which are derived from host innate immune responses, have broad-spectrum activity against Gram-positive and Gram-negative bacteria. Based on previous reports, antimicrobial proteins isolated from the small intestine of rabbits were found to display antimicrobial activity against *S. aureus*, *P. aeruginosa, E. coli,* and *C. albicans* [58].

### 6.5. Bacteriophages

Conventional antibiotic therapies are less useful for MRSA infections. Bacteriophage therapy is a novel and potential alternate therapy to overcome MRSA infections. It is recommended to combine the bacteriophage and antibiotic therapies to increase the efficiency of MRSA treatment [59]. Due to the complexity of bacterial community colonized in DFIs, a mixture of phages may require to target a wider range of the bacterial population. Anti-*Staphylococcus* phages have a broad range of activity; hence, two or three different phages are required to target different strains of *S. aureus.* In contrast, Gram-negative bacteria require a higher range of phages (>10) [60].

### 6.6. Negative-Pressure Wound Therapy

Negative-pressure wound therapy (NPWT) is a vacuum sealing method to control wound swelling and stimulate the formation of granulation tissue to speed up the wound healing process [61]. Based on a study conducted on 342 patients, wounds treated with NPWT healed in a shorter period of time, compared to traditional dressings [62]. NPWT can also be combined with the installation of effective antibacterial agents. It has been reported that patients with NPTW combined with antiseptic installation experienced shorter hospitalization, compared to NPTW without antiseptic installation [63]. However, due to insufficient published data in this regard, the productivity of this method has not been well documented yet.

### 6.7. Hyperbaric Oxygen Therapy

Hyperbaric oxygen therapy (HBOT) is the exposure of diabetic ulcers to a high concentration of oxygen in an adjustable atmospheric pressure. Based on previous findings, HBOT could decrease lower extremity amputation rate in patients with diabetic foot ulcers [64]. There is, however, no published data available to evaluate the effect of hyperbaric oxygen therapy on bone or tissue infections.

### 6.8. Stem Cell Therapy

Embryonic stem cells can transform into known differentiated cell types in the human body. Stem cell therapy can decrease the chance of amputation in diabetic people with ischaemic diabetic foot ulcers [65,66].

### 6.9. Off-Loading

Off-loading is primarily to keep the pressure off the affected area by using special boots, casts, or shoes to help the foot ulcers heal as quickly as possible. It also can reduce the risk of severe complications [57].

## 7. New Insights into DFIs Treatment Based on Molecular Findings

Despite many advances in the microbiology field, bacterial antibiotic resistance is still a major challenge, and little is known about the region, distribution, and diversity of resistance genes particularly for uncultivable bacteria, which constitute a large portion of the bacterial population and may play significant roles in infection progression. Several novel antibiotic resistance genes, including bleomycin, aminoglycosides, tetracycline, and β-lactam, have been identified using DNA sequence-based approaches [67]. However, DNA-based techniques only suggest the presence of a gene that may encode enzymes involved in resistant bacteria, but it does not necessarily confirm that the gene is functionally expressed or not. Metatranscriptomics can help us to reach this level of understanding and more important to study the host-pathogen co-transcriptome profile [47]. The study of slow-growing, fastidious, anaerobic, and unknown pathogens, which normally have been underestimated by culture-based methods will provide an early warning system necessary for modification and alteration of antibiotic therapy. The useful information from shotgun metagenomic sequencing and metatranscriptomic analysis can help researchers to anticipate the bacterial resistance profile before it emerges clinically. So far, our knowledge is limited to culturable bacteria and few studies conducted on 16S rRNA sequencing analysis on DFUs/DFIs. We need to apply new molecular approaches to track the transcriptional program involved in bacterial survival and how pathogens can subvert antimicrobial strategies, not only in culturable bacteria, but also in uncultivable microorganisms in DFUs/DFIs.

## 8. Conclusions

Continuous evaluation of the feet, proper use of antibiotics, surgical procedures, and multifaceted approaches emphasizing better diagnostic methods can prevent infection progression, and, more importantly, the risk of lower extremity amputation. Researchers and clinicians should be up-to-date and have an understanding of new methods of prevention, diagnosis, and treatment of DFIs.

There have been many studies on the bacteriology of DFUs/DFIs over the past decades with varying, and sometimes inconsistent results. These discrepancies might be due to demographical and geographical differences, various processes of sampling, human errors, sample size, and different bacterial identification methods used.

Even though significant advances have been made to manage DFIs, many unanswered questions about DFUs/DFIs microbiota exist. These questions will require help from new and advanced molecular technologies. A diverse range of studies has successfully evaluated transcriptional pathways involved in intramacrophage survival [68] and alteration of the bacterial transcriptomic profile in adaptation to human cells [69]. However, the host inflammatory responses and major bacterial metabolisms involved in DFIs have not been profiled yet. The output of dual meta-transcriptomic analysis or profiling of dynamic host-pathogen interactions offer strong prospects for further research on DFUs/DFIs.

It may be concluded that molecular approaches are more reliable than traditional methods in the study of DFUs/DFIs microbiota and can provide greater insights into DFI microbiology. However, due to the paucity of information, more investigation is needed to decide which method should be chosen as the primary identification tool.

## Figures and Tables

**Table 1:** Common bacterial species isolated from diabetic ulcers using different bacterial identification tools.

**Culture**								
**Reference**	**Specimen No.**	**Specimen**	**Predominant Aerobe**				**Predominant Facultative Anaerobe/Anaerobe**	
Slater, Lazarovitch et al., 2004 [9]	60	Swab, biopsy	*S. aureus* (50%), Coagulase-negative *Staphylococci* (38%), *Enterococcus* (20%), *Diphtheroid* spp. (10%), *Acinetobacter* (7%)				*Streptococcus* (25%), *Proteus* (23%), *Escherichia coli* (17%), *Klebsiella* (12%), *Enterobacter* (10%), *Pseudomonas* (10%), *Citrobacter* (8%), *Anaerobic cocci* (13%), *Anaerobic rods* (3%), *Bacteroides* (3%)	
Citron, Goldstein et al., 2007 [23]	454	Curettage, Biopsy	*Streptococcus* spp. (15.5%), *Staphylococcus* spp. (15.3%), *oxacillin-susceptible S. aureus* (14.3%), *oxacillin-resistant S. aureus* (4.4%), *Coagulase negative, Enterococcus* spp. (13.5%), *Enterobacteriaceae family* (12.8%), *Corynebacterium* spp. (10.1%), *P. aeruginosa* (3.5%)				*Gram-positive cocci* (45.2%), *Finegoldia magna* (37.4), *Prevotella* spp. (13.6%), *Porphyromonas* spp. (11.3%), *Bacteroides fragilis group* (10.2%)	
Al Benwan, Al Mulla et al., 2012 [21]	440	Curettage	*P. aeruginosa* (17.4%), *S. aureus* (11.8%), *methicillin-resistant S. aureus* (7.7%)				*Enterobacteriaceae* (28.5%), *anaerobic Gram-negative organisms* (10.8%), *Enterococcus* spp. (7%)	
Olowu, Eyaufe et al., 2013 [42]	150	Swab	*S. aureus* (38%), *P. aeruginosa* (8%)				*Escherichia coli* (24%), *Proteus* spp. (20%), *Klebsiella* spp. (10%)	
Djahmi, Messad et al., 2013 [43]	128	Aspiration, Biopsy, Swab	*S. aureus* (30.7%), Coagulase-negative *Staphylococcus* (11.2%), *P. aeruginosa* (8.3%), *Morganella morganii* (5.4%), *Acinetobacter baumannii* (2.9%)				*Proteus mirabilis* (12.6%), *Klebsiella pneumoniae* (11.2%), *Escherichia coli* (7.2%), *Enterobacter cloacae* (3.2%), *Enterococcus faecalis* (2.5%), *Proteus vulgaris* (2.5%), *Streptococcus* spp. (0.7%), *Providencia stuartii* (0.7%), *Klebsiella oxytoca* (0.4%), *Citrobacter* spp. (0.4%)	
Olowu, Eyaufe et al., 2013 [42]	86	Swab, Biopsy	*Enterococcus* spp. (27%), *Staphylococcus: CONs* (22%), *Staphylococcus: COPs* (7%), *Diphtheroid* spp. (2%), *Acinetobacter baumannii* (1%), *Acinetobacter lwoffii* (1%), *Morganella morganii* (1%), *S. maltophilia* (1%)				*Escherichia coli* (20%), *Bacillus* (3%), *Proteus* spp. (3%), *P. aeruginosa* (2%), *Klebsiella pneumoniae* (1%), *β-hemolytic streptococci* (1%), *Serratia liquefaciens* (1%), *Enterobacter gergoviae* (1%)	
Miyan, Fawwad et al., 2017 [24]	342	Bone, Pus, Biopsy	*S. aureus* (20.67%), *Pseudomonas* (13.54%), *Acinetobacter baumannii* (5.24%), *Morganella morganii* (1.75%), *Coagulase-negative staphylococci* (0.73%), *Enterococus* species (0.44%)				*Escherichia coli* (15.72%), *Klebsiella pneumoniae* (13.54%), *Proteus mirabilis* (12.81%), *Proteus species* (6.11%), *Proteus vulgaris* (4.37%), *Streptococcus* species (1.89%), *Enterobacter* Species (1.75%), *Citrobacter* spp. (1.46%)	
**Culturomics**								
**Reference**	**Specimen No.**	**Specimen**	**Predominant Aerobe**				**Predominant Facultative Anaerobe/Anaerobe**	
Jneid, Cassir et al., 2018 [20]	43	Swab, debris	*S. aureus* (52.8%), *Staphylococcus lugdunensis* (18.7%), *S. epidermidis* (11.3%)				*Enterococcus faecalis* (45.2%), *Enterobacter cloacae* (22.6%), *Proteus mirabilis* (11.3%), *Finegoldia magna* (9.4%)	
**16S rRNA Sequencing**								
**Reference**	**Specimen No.**	**Specimen**	**DNA Extraction Method**	**Target Region**	**Sequencing Platform**	**Read per Sample**	**Predominant Aerobe**	**Predominant Facultative Anaerobe/Anaerobe**
Ge, MacDonald et al., 2002 [44]	39	Biopsy	Bead-beating combined with Mo Bio PowerBiofilm kit	V1–V4	Illumina MiSeq	31,452	The most commonly found species: *Staphylococcus* spp., *Corynebacterium* spp., *Acinetobacter* spp.	The most commonly found species: *Streptococcus* spp. *Anaerococcus* spp., *Finegoldia* spp., *Peptoniphilus* spp.
Dowd, Wolcott et al., 2008	40	Debridement	Qiagen TissueLyser combined with QIAamp DNA Mini Kit	-	bTEFAP	-	*Corynebacterium* spp.:14.4%, *Streptococcus* spp.: 36.5%, *Pseudomonas* spp.: 14.5%, *Staphylococcus* spp.: 8.3%	*Bacteroides* spp.:24.2%, *Peptoniphilus* spp.: 13.6%, *Serratia* spp.: 21.4%, *Finegoldia* spp.: 6.7%, *Anaerococcus* spp.: 7.7%, *Prevotella* spp.: 7.4%, *Peptostreptococcus* spp.: 8.7%, *Porphyromonas* spp.:7%, *Actinomyces* spp.: 5.7%, *Varibaculum* spp.: 9%, *Fusobacterium* spp.:5.6%, *Citrobacter* spp.: 9.5%, *Rothia* spp.: 5.8%
Gardner, Hillis et al., 2013 [33]	52	Swab	Bead-beating combined with a QIAampDNA Mini Kit	V1–V3	Roche 454 FLX Titanium	5634	*S. aureus*: 96.5%, *S. epidermidis*: 0.4%), *Actinobacteria* (14%)	*Proteobacteria* (9.8%), *Bacteroidetes* (7.3%), and *Fusobacteria* (1.4%)
Smith, Collier et al., 2016 [31]	16	Swab	Bead-beating combined with a QIAamp DNA Mini Kit	V4	Illumina Hiseq 2500	110,447	New ulcers: *Staphylococcus* (31.25%), *Corynebacterium* (25%) Recurrent ulcers: *Corynebacterium* (31.25%)	New ulcers: *Peptoniphilus* (37.5 %), *Anaerococcus* (31.25%) Recurrent ulcers: *Peptoniphilus* (12%), *Anaerococcus* (25%)
Wolcott, Hanson et al., 2016 [35]	910	Biopsy	Bead-beating combined with High PurePCR Template Preparation Kit	V1–V3	Roche 454	-	*S. epidermidis* (38%), *S. aureus* (33%), *S. haemolyticus* (21%), *S. lugdunensis* (18%), *Stenotrophomonas maltophilia* (16%), *P. aeruginosa* (14%), *Corynebacterium* (13%), *Corynebacterium striatum* (12%), *Staphylococcus pettenkoferi* (9%), *Acinetobacter baumannii* (5%), *Corynebacterium jeikeium* (5%) *Ralstonia pickettii*	*Finegoldia magna* (25%), *Enterococcus faecalis* (17%), *Anaerococcus vaginalis* (13%), *Streptococcus agalactiae* (10%), *Enterobacter hormaechei* (9%), *Prevotella bivia* (9%), *Delftia acidovorans* (5%), *Serratia nematodiphila* (5%), *Proteus mirabilis* (4%), *Streptococcus salivarius, Fusobacterium nucleatum, Bacteroides fragilis, Flavobacterium succinicans*
Gardiner, Vicaretti et al., 2017 [45]	257	Swab, Biopsy	Bead-beating combined with BioStic DNA extraction kit	V4	Illumina Miseq	-	The most commonly found species: *Staphylococcus*, followed by *Acinetobacter*, *Corynebacterium, unclassified Enterobacteriacea*.	
Loesche, Gardner et al., 2017 [46]	100	Swab	Bead-beating combined with a QIAamp DNA Mini Kit	V1–V3	Illumina Miseq	22,070	*Staphylococcus* (22.77%, *S. aureus* (13.3%), *Staphylococcus pettenkoferi* (5.3%)), *Streptococcus* (11.98%), *Corynebacterium* (11.46%)	*Anaerococcus* (7%)
**Metagenomicss**								
**Reference**	**Specimen No.**	**Specimen**	**DNA Extraction Method**	**Bacterial DNA Enrichment**	**Sequencing Platform**	**Read per Sample**	**Predominant Aerobe**	**Predominant Facultative Anaerobe/Anaerobe**
Kalan, Meisel et al., 2018 [40]	100	Swab	Bead-beating combined with a QIAamp DNA Mini Kit	NEBNext Microbiome DNA Enrichment kit	HiSeq 4000	144,416,914	*Staphylococcus* (18.95%: *S. aureus, S. aureus 7372, S. pettenkoferi, S. epidermidis, S. simulans, S. lugdunensis*), *Corynebacterium* (14.64%, *Corynebacterium striatum, C. jeikeium, C. amycolatum, C. pseudogenitalium, C. tuberculostearicum, C. resistens*), *Pseudomonas* (9.37%, *P. aeruginosa, P. alcaliphila*), *Streptococcus* (7.32%, *S. agalactiae, S. dysgalactiae, S. anginosus*)	*Anaeroccocus, Porphyromonas, Prevotella, Veillonella* spp.

## References

[1] Commons R.J., Raby E., Athan E., Bhally H., Chen S., Guy S., Ingram P.R., Lai K., Lemoh C., Lim L.-L. (2018). Managing diabetic foot infections: A survey of Australasian infectious diseases clinicians. J. Foot Ankle Res..

[2] IDF diabetes atlas—8th edition. http://www.diabetesatlas.org/.

[3] Raghav A., Khan Z.A., Labala R.K., Ahmad J., Noor S., Mishra B.K. (2018). Financial burden of diabetic foot ulcers to world: A progressive topic to discuss always. Ther. Adv. Endocrinol. Metab..

[4] Rhoads D.D., Wolcott R.D., Sun Y., Dowd S.E. (2012). Comparison of culture and molecular identification of bacteria in chronic wounds. Int. J. Mol. Sci..

[5] Lipsky B.A. (2007). Diabetic foot infections: Microbiology made modern?: Array of hope. Diabetes Care.

[6] Lipsky B.A., Berendt A.R., Cornia P.B., Pile J.C., Peters E.J., Armstrong D.G., Deery H.G., Embil J.M., Joseph W.S., Karchmer A.W. (2012). 2012 Infectious Diseases Society of America clinical practice guideline for the diagnosis and treatment of diabetic foot infections. Clin. Infect. Dis..

[7] Lipsky B.A., Aragón-Sánchez J., Diggle M., Embil J., Kono S., Lavery L., Senneville É., Urbančič-Rovan V., Van Asten S., Peters E.J. (2016). IWGDF guidance on the diagnosis and management of foot infections in persons with diabetes. Diabetes Metab. Res. Rev..

[8] Pellizzer G., Strazzabosco M., Presi S., Furlan F., Lora L., Benedetti P., Bonato M., Erle G., De Lalla F. (2001). Deep tissue biopsy vs. superficial swab culture monitoring in the microbiological assessment of limb-threatening diabetic foot infection. Diabet. Med..

[9] Slater R., Lazarovitch T., Boldur I., Ramot Y., Buchs A., Weiss M., Hindi A., Rapoport M. (2004). Swab cultures accurately identify bacterial pathogens in diabetic foot wounds not involving bone. Diabet. Med..

[10] Mutluoglu M., Uzun G., Turhan V., Gorenek L., Ay H., Lipsky B.A. (2012). How reliable are cultures of specimens from superficial swabs compared with those of deep tissue in patients with diabetic foot ulcers?. J. Diabetes Its Complicat..

[11] Huang Y., Cao Y., Zou M., Luo X., Jiang Y., Xue Y., Gao F. (2016). A comparison of tissue versus swab culturing of infected diabetic foot wounds. Int. J. Endocrinol..

[12] Senneville E., Melliez H., Beltrand E., Legout L., Valette M., Cazaubie M., Cordonnier M., Caillaux M., Yazdanpanah Y., Mouton Y. (2006). Culture of percutaneous bone biopsy specimens for diagnosis of diabetic foot osteomyelitis: Concordance with ulcer swab cultures. Clin. Infect. Dis..

[13] Elamurugan T., Jagdish S., Kate V., Parija S.C. (2011). Role of bone biopsy specimen culture in the management of diabetic foot osteomyelitis. Int. J. Surg..

[14] Nelson E.A., Wright-Hughes A., Brown S., Lipsky B.A., Backhouse M., Bhogal M.S., Ndosi M., Reynolds C., Sykes G., Dowson C. (2016). Concordance in diabetic foot ulceration: A cross-sectional study of agreement between wound swabbing and tissue sampling in infected ulcers. Health Technol. Assess..

[15] Jneid J., Lavigne J., La Scola B., Cassir N. (2017). The diabetic foot microbiota: A review. Hum. Microbiome J..

[16] Abdulrazak A., Bitar Z.I., Al-Shamali A.A., Mobasher L.A. (2005). Bacteriological study of diabetic foot infections. J. Diabetes Its Complicat..

[17] El-Tahawy A. (2000). Bacteriology of diabetic foot. Saudi Med J..

[18] Lee J.S., Son S.T., Han S.-K. (2017). Risk factors of methicillin-resistant Staphylococcus aureus and Pseudomonas infection in diabetic foot ulcers in Korea. J. Wound Manag. Res..

[19] Tascini C., Piaggesi A., Tagliaferri E., Iacopi E., Fondelli S., Tedeschi A., Rizzo L., Leonildi A., Menichetti F. (2011). Microbiology at first visit of moderate-to-severe diabetic foot infection with antimicrobial activity and a survey of quinolone monotherapy. Diabetes Res. Clin. Pract..

[20] Jneid J., Cassir N., Schuldiner S., Jourdan N., Sotto A., Lavigne J.-P., La Scola B. (2018). Exploring the microbiota of diabetic foot infections with culturomics. Front. Cell. Infect. Microbiol..

[21] Al Benwan K., Al Mulla A., Rotimi V.O. (2012). A study of the microbiology of diabetic foot infections in a teaching hospital in Kuwait. J. Infect. Public Health.

[22] Yao Y., Sturdevant D.E., Villaruz A., Xu L., Gao Q., Otto M. (2005). Factors characterizing Staphylococcus epidermidis invasiveness determined by comparative genomics. Infect. Immun..

[23] Citron D.M., Goldstein E.J., Merriam C.V., Lipsky B.A., Abramson M.A. (2007). Bacteriology of moderate-to-severe diabetic foot infections and in vitro activity of antimicrobial agents. J. Clin. Microbiol..

[24] Miyan Z., Fawwad A., Sabir R., Basit A. (2017). Microbiological pattern of diabetic foot infections at a tertiary care center in a developing country. Age Years.

[25] Sánchez-Sánchez M., Cruz-Pulido W.L., Bladinieres-Cámara E., Alcalá-Durán R., Rivera-Sánchez G., Bocanegra-García V. (2017). bacterial prevalence and antibiotic resistance in clinical isolates of diabetic foot ulcers in the Northeast of Tamaulipas, Mexico. Int. J. Low. Extrem. Wounds.

[26] Viswanathan V., Jasmine J.J., Snehalatha C., Ramachandran A. (2002). Prevalence of pathogens in diabetic foot infection in South Indian type 2 diabetic patients. J. Assoc. Physicians India.

[27] Lagier J.-C., Hugon P., Khelaifia S., Fournier P.-E., La Scola B., Raoult D. (2015). The rebirth of culture in microbiology through the example of culturomics to study human gut microbiota. Clin. Microbiol. Rev..

[28] Woo P., Lau S., Teng J., Tse H., Yuen K.-Y. (2008). Then and now: Use of 16S rDNA gene sequencing for bacterial identification and discovery of novel bacteria in clinical microbiology laboratories. Clin. Microbiol. Infect..

[29] Lavigne J.-P., Sotto A., Dunyach-Remy C., Lipsky B.A. (2015). New Molecular techniques to study the skin microbiota of diabetic foot ulcers. Adv. Wound Care.

[30] Ranjan R., Rani A., Metwally A., McGee H.S., Perkins D.L. (2016). Analysis of the microbiome: Advantages of whole genome shotgun versus 16S amplicon sequencing. Biochem. Biophys. Res. Commun..

[31] Smith K., Collier A., Townsend E.M., O’Donnell L.E., Bal A.M., Butcher J., Mackay W.G., Ramage G., Williams C. (2016). One step closer to understanding the role of bacteria in diabetic foot ulcers: Characterising the microbiome of ulcers. BMC Microbiol..

[32] Suryaletha K., John J., Radhakrishnan M.P., George S., Thomas S. (2018). Metataxonomic approach to decipher the polymicrobial burden in diabetic foot ulcer and its biofilm mode of infection. Int. Wound J..

[33] Gardner S.E., Hillis S.L., Heilmann K., Segre J.A., Grice E.A. (2013). The neuropathic diabetic foot ulcer microbiome is associated with clinical factors. Diabetes.

[34] Dowd S.E., Sun Y., Secor P.R., Rhoads D.D., Wolcott B.M., James G.A., Wolcott R.D. (2008). Survey of bacterial diversity in chronic wounds using Pyrosequencing, DGGE, and full ribosome shotgun sequencing. BMC Microbiol..

[35] Wolcott R.D., Hanson J.D., Rees E.J., Koenig L.D., Phillips C.D., Wolcott R.A., Cox S.B., White J.S. (2016). Analysis of the chronic wound microbiota of 2,963 patients by 16S rDNA pyrosequencing. Wound Repair Regen..

[36] Malone M., Johani K., Jensen S.O., Gosbell I.B., Dickson H.G., Hu H., Vickery K. (2017). Next generation DNA sequencing of tissues from infected diabetic foot ulcers. EBioMedicine.

[37] Dowd S.E., Wolcott R.D., Sun Y., McKeehan T., Smith E., Rhoads D. (2008). Polymicrobial nature of chronic diabetic foot ulcer biofilm infections determined using bacterial tag encoded FLX amplicon pyrosequencing (bTEFAP). PLoS ONE.

[38] Wilmes P., Bond P.L. (2006). Metaproteomics: Studying functional gene expression in microbial ecosystems. Trends Microbiol..

[39] Thomas T., Gilbert J., Meyer F. (2012). Metagenomics-a guide from sampling to data analysis. Microb. Inform. Exp..

[40] Kalan L., Meisel J.S., Loesche M.A., Horwinski J., Soaita I., Chen X., Gardner S.E., Grice E.A. (2018). The microbial basis of impaired wound healing: Differential roles for pathogens, “bystanders”, and strain-level diversification in clinical outcomes. bioRxiv.

[41] Větrovský T., Baldrian P., Morais D. (2018). SEED 2: A user-friendly platform for amplicon high-throughput sequencing data analyses. Bioinformatics.

[42] Olowu S., Eyaufe A., Edomwonyi E., Victoria O., Imuetinyan E., Adesuwa E. (2013). Aerobic bacteria associated with diabetic wounds in patients attending clinic in a rural community In Nigeria. Glob. Res. J. Microbiol..

[43] Djahmi N., Messad N., Nedjai S., Moussaoui A., Mazouz D., Richard J.L., Sotto A., Lavigne J.P. (2013). Molecular epidemiology of Staphylococcus aureus strains isolated from inpatients with infected diabetic foot ulcers in an Algerian University Hospital. Clin. Microbiol. Infect..

[44] Ge Y., MacDonald D., Hait H., Lipsky B., Zasloff M., Holroyd K. (2002). Microbiological profile of infected diabetic foot ulcers. Diabet. Med. A J. Br. Diabet. Assoc..

[45] Gardiner M., Vicaretti M., Sparks J., Bansal S., Bush S., Liu M., Darling A., Harry E., Burke C.M. (2017). A longitudinal study of the diabetic skin and wound microbiome. PeerJ.

[46] Loesche M., Gardner S.E., Kalan L., Horwinski J., Zheng Q., Hodkinson B.P., Tyldsley A.S., Franciscus C.L., Hillis S.L., Mehta S. (2017). Temporal stability in chronic wound microbiota is associated with poor healing. J. Investig. Dermatol..

[47] Moran M.A. (2009). Metatranscriptomics: Eavesdropping on complex microbial communities. Microbe.

[48] Kukurba K.R., Montgomery S.B. (2015). RNA sequencing and analysis. Cold Spring Harb. Protoc..

[49] Sotto A., Richard J.-L., Jourdan N., Combescure C., Bouziges N., Lavigne J.-P. (2007). Miniaturized Oligonucleotide Arrays A new tool for discriminating colonization from infection due to Staphylococcus aureus in diabetic foot ulcers. Diabetes Care.

[50] Sotto A., Lina G., Richard J.-L., Combescure C., Bourg G., Vidal L., Jourdan N., Etienne J., Lavigne J.-P. (2008). Virulence potential of Staphylococcus aureus strains isolated from diabetic foot ulcers: A new paradigm. Diabetes Care.

[51] Abbas M., Uçkay I., Lipsky B.A. (2015). In diabetic foot infections antibiotics are to treat infection, not to heal wounds. Expert Opin. Pharmacother..

[52] Zenelaj B., Bouvet C., Lipsky B.A., Uçkay I. (2014). Do diabetic foot infections with methicillin-resistant staphylococcus aureus differ from those with other pathogens?. Int. J. Low. Extrem. Wounds.

[53] Teterycz D., Ferry T., Lew D., Stern R., Assal M., Hoffmeyer P., Bernard L., Uçkay I. (2010). Outcome of orthopedic implant infections due to different staphylococci. Int. J. Infect. Dis..

[54] Gottrup F., Apelqvist J., Bjarnsholt T., Cooper R., Moore Z., Peters E.J.G., Probst S. (2014). Antimicrobials and Non-Healing Wounds. Evidence, controversies and suggestions—Key messages. J. Wound Care.

[55] Roberts A.D., Simon G.L. (2012). Diabetic foot infections: The role of microbiology and antibiotic treatment. Semin. Vasc. Surg..

[56] Lipsky B.A., Armstrong D.G., Citron D.M., Tice A.D., Morgenstern D.E., Abramson M.A. (2005). Ertapenem versus piperacillin/tazobactam for diabetic foot infections (SIDESTEP): Prospective, randomised, controlled, double-blinded, multicentre trial. Lancet.

[57] Uçkay I., Aragón-Sánchez J., Lew D., Lipsky B. (2015). Diabetic foot infections: What have we learned in the last 30 years?. Int. J. Infect. Dis..

[58] Xia X., Cheng L., Zhang S., Wang L., Hu J. (2018). The role of natural antimicrobial peptides during infection and chronic inflammation. Antonie van Leeuwenhoek.

[59] Zhabiz G., Omar B., Donald Gene P. (2014). Bacteriophage therapy: A potential solution for the antibiotic resistance crisis. J. Infect. Dev. Ctries..

[60] Aragón-Sánchez J., Lipsky B.A. (2018). Modern management of diabetic foot osteomyelitis. The when, how and why of conservative approaches. Expert Rev. Anti Infect. Ther..

[61] Guffanti A. (2014). Negative pressure wound therapy in the treatment of diabetic foot ulcers. J. Wound Ostomy Cont. Nurs. Off. Publ. Wound Ostomy Cont. Nurses Soc..

[62] Blume P.A., Walters J., Payne W., Ayala J., Lantis J. (2008). Comparison of negative pressure wound therapy using vacuum-assisted closure with advanced moist wound therapy in the treatment of diabetic foot ulcers: A multicenter randomized controlled trial. Diabetes Care.

[63] Kim P.J., Attinger C.E., Oliver N., Garwood C., Evans K.K., Steinberg J.S., Lavery L.A. (2015). Comparison of outcomes for normal saline and an antiseptic solution for negative-pressure wound therapy with instillation. Plast. Reconstr. Surg..

[64] Löndahl M., Katzman P., Nilsson A., Hammarlund C. (2010). Hyperbaric oxygen therapy facilitates healing of chronic foot ulcers in patients with diabetes. Diabetes Care.

[65] Zuk P.A., Zhu M., Mizuno H., Huang J., Futrell J.W., Katz A.J., Benhaim P., Lorenz H.P., Hedrick M.H. (2001). Multilineage cells from human adipose tissue: Implications for cell-based therapies. Tissue Eng..

[66] Li X.-Y., Zheng Z., Li X.-Y., Guo J., Zhang Y., Li H., Wang Y.-W., Ren J.F., Wu Z.-B. (2013). Treatment of foot disease in patients with type 2 diabetes mellitus using human umbilical cord blood mesenchymal stem cells: Response and correction of immunological anomalies. Curr. Pharm. Des..

[67] Schmieder R., Edwards R. (2011). Insights into antibiotic resistance through metagenomic approaches. Future Microbiol..

[68] Mavromatis C., Bokil N.J., Totsika M., Kakkanat A., Schaale K., Cannistraci C.V., Ryu T., Beatson S.A., Ulett G.C., Schembri M.A. (2015). The co-transcriptome of uropathogenic Escherichia coli-infected mouse macrophages reveals new insights into host–pathogen interactions. Cell. Microbiol..

[69] Aprianto R., Slager J., Holsappel S., Veening J.-W. (2016). Time-resolved dual RNA-seq reveals extensive rewiring of lung epithelial and pneumococcal transcriptomes during early infection. Genome Biol..

